# Dietary energy enhances conception in Holstein heifers via interactions with rumen microbiota

**DOI:** 10.5713/ab.25.0141

**Published:** 2025-07-11

**Authors:** Xusheng Hao, Taiping Wu, Xia Li, Qiuyue He, Yulong Qin, Nan Zhang, Haotian Yu, Yujun Jiang, Feng Gao

**Affiliations:** 1 College of Animal Science, Inner Mongolia Agricultural University, Hohhot, China; 2 Inner Mongolia Shengmu High-tech Animal Husbandry Co., Ltd., Hohhot, China

**Keywords:** Energy Density, Gonadotropin Axis, Holstein Heifers, Metabolic Interaction, Rumen Microbiome

## Abstract

**Objective:**

This study aimed to elucidate how dietary energy levels regulate the hypothalamic-pituitary-gonadal axis in Holstein heifers during initial breeding, with a focus on rumen microbiota-host interactions.

**Methods:**

Forty-four pubertal heifers (398.96±6.56 kg BW, 12.72±0.02 months) were stratified by body condition score and estrous cyclicity, then randomly allocated to control (CON, 8.64 MJ/kg DM NE_L_) or high-energy (HE, 9.50 MJ/kg DM NE_L_) diets (n = 22/group). Although practical constraints limited pen replication, we implemented rigorous matching procedures: Pens were matched for surface area (120 m^2^), feed bunk space (0.8 m/head) and growth performance, serum biochemical/immune/antioxidant markers, reproductive hormones, rumen fermentation parameters, microbiota, and metabolome profiles were analyzed.

**Results:**

The HE group exhibited elevated gonadotropins (follicle-stimulating hormone, luteinizing hormone [LH]) and prolactin (PRL), indicating enhanced hypothalamic-pituitary activity. Serum triglycerides increased, while immune markers showed the altered state of immunoregulation characterized by significant increases in interleukin (IL)-2 and IL-6, reductions in IL-4, and decreases in tumor necrosis factor-α and interferon-γ. Antioxidant capacity improved with lower malondialdehyde levels. Rumen pH decreased, accompanied by elevated total volatile fatty acid, bacterial crude protein, acetic acid, propionic acid, butyric acid, and valeric acid concentrations. Microbial shifts included *Treponema* and *Prevotellaceae_UCG_003* showing positive correlations with PRL and LH, while *Ruminococcus* was associated with acetyl-CoA precursors through enriched pyruvate metabolism.

**Conclusion:**

HE diets (9.50 MJ/kg NE_L_) enhance hypothalamic-pituitary signaling and rumen fermentation efficiency, advancing first-service conception rates by 15% (55% vs. 70%) in pasture-based systems. This strategy optimizes reproductive management in intensive dairy operations through microbiota-driven metabolic modulation.

## INTRODUCTION

As the mainstay of the global dairy industry, the performance of Holstein heifers is directly related to the economic efficiency of the dairy industry. In northern China’s pastoral regions (e.g., Inner Mongolia), Holstein heifers reared under traditional forage-based diets often exhibit delayed puberty and low first-service conception rates (<50%), leading to prolonged calving intervals and economic losses. Given these challenges, this study hypothesizes that graded dietary energy levels can modulate the hypothalamic-pituitary-gonadal axis through rumen microbiota-metabolome interactions, thereby optimizing reproductive outcomes and growth performance. Local farmers increasingly adopt high-concentrate diets to accelerate reproductive maturity, yet the optimal energy density for balancing rumen health and endocrine function remains unclear. This research employs a multi-omics approach—integrating rumen microbiome and metabolomics—to explore how dietary energy levels modulate rumen microbiota and metabolic pathways. Specifically, we aim to determine the optimal energy density needed to balance rumen health and endocrine function, with broader implications for precision feeding strategies in semi-arid grazing systems. Dietary energy critically regulates ruminant physiology, directly impacting production, health, and nutrient utilization efficiency. Scientific regulation of dietary energy levels is paramount for optimizing ruminant feeding strategies and enhancing farming efficiency [1,2].

Optimal dietary energy levels stimulate animal growth, facilitate early service, reduce calving intervals, and enhance breeding benefits. Most large-scale cattle farms set the age at first service at 13–14 months, with some advancing to 12 months [3]. Early heifer service, pregnancy, parturition, and lactation offer economic benefits, yet premature pregnancy may divert energy from somatic growth [4–6], potentially impairing mammary gland development and reproductive organ maturation. This energy diversion can result in suboptimal milk synthesis capacity in subsequent lactations, ultimately reducing milk yield. Research indicates that the optimal first service age of dairy cows is positively correlated with productive lifespan. The transition to more refined and efficient dairy practices necessitates a comprehensive exploration of how varying energy levels during the critical initial service period influence not only reproductive outcomes but also other physiological systems.

The rumen microbiota, a critical component of ruminant physiology, is closely linked to nutrient utilization and metabolic processes. The dietary composition influences the structure and function of the rumen microbiota, the molar concentration and proportion of volatile fatty acids (VFAs), and the gastrointestinal tract morphology [7]. With the intensification of dairy farming, high-concentrate diets have increasingly replaced traditional forage-based regimens. Such dietary shifts alter rumen microecosystem equilibrium and gastrointestinal tract development, which in turn modulate key performance traits including growth efficiency and fecundity [8]. Furthermore, research indicates that variations in dietary metabolic energy intake can impact serum metabolic hormones and oxidative stress markers [9]. For instance, high-concentrate diets have been shown to alter gastrointestinal tract development, influencing nutrient absorption and utilization efficiency [10]. Similarly, studies have demonstrated that dietary energy levels can affect oxidative stress markers, with potential implications for animal health and productivity [11].

This study employs a multi-omics approach to systematically evaluate how graded dietary energy levels during the first service period affect Holstein heifers’ growth performance, reproductive competence, serum metabolic profiles, antioxidant status, rumen fermentation kinetics, and microbial community assembly. Findings from this study will provide actionable insights for formulating precision feeding strategies that balance early reproductive goals with long-term lactation potential in commercial herds.

## MATERIALS AND METHODS

### Ethical statement

All experimental protocols in this study were approved by the Animal Care and Ethics Committee of Inner Mongolia Agricultural University (approval number NND 2021104), and the animals were maintained following the institutional guidelines for the care and use of laboratory animals in China.

### Experimental design, animals, and management

This experiment was conducted in Shengmu Ranch, located in Inner Mongolia, China. Methodological implementation proceeded through a rigorous selection of bovine subjects meeting predefined criteria (398.96±6.56 kg, 12.72±0.02 months old) from the designated ranch cohort, yielding 45 qualified individuals. Due to population size constraints preventing uniform group distribution, subjects underwent randomized allocation (n = 22/group) using computer-generated permutation sequences. The two groups were a control group (CON, base diet) and a high-energy group (HE, high-energy diet). Although practical constraints limited pen replication, we implemented rigorous matching procedures: Pens were matched for surface area (120 m^2^), and feed bunk space (0.8 m/head). Biological sampling employed a stratified design with quintuplicate replicates per experimental condition, each comprising a random selection of 2 subjects from 5-animal cohorts. This configuration statistically ensured reproducibility while maintaining operational feasibility under field constraints. The composition of the diet is shown in Table 1. The total mixed ration (TMR) was fed twice daily (10:00 a.m. and 5:00 p.m., respectively). All cows ad libitum access to water, and were allowed to move freely within the designated area.

A 7-day preliminary phase preceded the 21-day main trial. All biospecimen collections were strategically timed to exclude phases of the bovine estrous cycle, with rigorous exclusion of samples obtained during follicular and luteal phases. On the 1st and 21st days, before the morning feeding, 10 cows per group were randomly selected to have their body dimensions measured and body weights calculated. Moreover, on the 21st day, before the morning feeding, blood samples were collected from the jugular vein using disposable vacuum ordinary blood collection tubes (KWS Kangweishi). Rumen fluid collection occurred post-morning feeding 2 h using a specialized gastric tube apparatus. Estrus detection was systematically conducted through multiparametric assessment protocols. Natural observation: Observed no fewer than 3 times daily, focusing on whether the cow accepts mounting from other cows, and the quantity and appearance of mucous secretions. Checked follicle development when necessary. Estrous observation times were 6:00, 12:00, 19:00, and 23:00. Tail-base marking: A mark 15 cm long and 3–5 cm wide made daily with paint at the tail base of cows in the breeding group. When paint distribution became irregular, observed hair and external genitalia. After estrus confirmation, maintained estrus records; Gynecological examination: Cows with reproductive diseases were excluded from estrous synchronization and breeding. For cows that did not exhibit estrus recurrence within 33–39 days after breeding, a B-ultrasound instrument (BXL-V60; Zhengzhou Boxianglai Electronic Technology) was employed for pregnancy testing. Pregnancy is diagnosed when the mean follicle diameter reaches approximately 14–15 mm or a follicle wave emerges. During the entire experimental period, the number of estrus occurrences, returns to estrus, miscarriages, cases of empty pregnancy, and successful pregnancies for each cow were meticulously recorded. Subsequently, the estrus rate, conception rate, and pregnancy rate for each group were computed and analyzed.

### Sample collection and measurement methods

#### Growth performance

Individual body weights were recorded pre- and post-trial using a digital scale to calculate average daily gain (ADG). Feed intake was monitored at the group level to determine the average daily feed intake (ADFI) and feed-to-gain (F/G) ratio.

#### Reproductive performance

The breeding data of Holstein Heifers were recorded, and the estrus rate (number of cows showing estrus/number of cattle to be bred) conception rate (number of pregnancies/number of cattle to be bred), re-estrus rate (number of re-estrus/number of cattle to be bred) pregnancy rate (number of pregnancy/number of cattle to be bred) were calculated.

#### Serum biochemical, immune, antioxidant, and reproductive hormone indices

Venous blood samples (10 mL) were obtained via jugular venipuncture using disposable needles and collected into vacuum tubes. After centrifugation at 1,200×g for 15 min, the upper serum layer was harvested. All samples underwent hemolysis assessment; only those with yellow and clear serum were retained. They were then aliquoted and stored at –80°C for long-term preservation to ensure sample integrity for subsequent experiments.

### Cytokine analysis

Levels of tumor necrosis factor-α (TNF-α), interferon-γ (IFN-γ), interleukin-2 (IL-2), interleukin-4 (IL-4), and interleukin-6 (IL-6) were assessed using enzyme-linked immunosorbent assays (ELISA), performed according to the manufacturer’s instructions.

### Antioxidant parameters

Total antioxidant capacity (T-AOC), superoxide dismutase (SOD), glutathione peroxidase (GSH-Px), catalase (CAT), and malondialdehyde (MDA) were quantified using commercial assay kits.

### Biochemical parameters

Aspartate aminotransferase (AST), alanine aminotransferase (ALT), alkaline phosphatase (ALP), albumin (ALB), triglycerides (TG), total protein (TP), lactate dehydrogenase (LDH), and glucose (GLU) were determined using a fully automated biochemical analyzer (Hitachi 3100).

### Reproductive hormones

Progesterone (Prog), estradiol (E2), luteinizing hormone (LH), follicle-stimulating hormone (FSH), and serum prolactin (PRL) were measured by radioimmunoassay using an automated system (XH-6020 Gamma Counter).

Before each detection, calibration was performed using the quality-control serum provided in the kit. A calibration curve was generated based on the counts per minute of standard samples, and assay values were calculated accordingly. To ensure quality control, each assay batch included standard hormones, quality-control samples with known hormone concentrations, and the to-be-tested samples. The above kits were purchased from Nanjing Jiancheng Bioengineering Institute. All measurements were performed in duplicate with parallel calibration using kit-supplied standards. The reproducibility criteria for data results required compliance with precision thresholds of intra-assay coefficients of variation (CV) <5% and inter-assay (CV) <10%.

### Rumen fermentation parameters

Ruminal fluid (100 mL) was collected using a bovine stomach tube cannula. The initial 50 mL aliquot was discarded to prevent salivary contamination. The remaining fluid was filtered through four layers of sterile medical gauze and subsequently aliquoted into 15 mL centrifuge tubes. Rumen fluid pH was measured immediately after collection using a calibrated pH meter (PHS-3C). The concentration of NH_3_-N was determined according to the colorimetric method. The composition and content of VFAs were determined by a Shimadzu (GC-2010) gas chromatograph. In the determination of BCP concentration, the rumen microorganisms in the sample were separated by differential centrifugation, the cell wall was broken by ultrasonic instrument, then the protein in the broken cell wall was stained by Coomassie brilliant blue method, and the BCP concentration was determined by colorimetric method at 595 nm by an enzyme-labeling analyzer [12].

### High-throughput 16S ribosomal RNA gene sequencing

Total genomic DNA was extracted from ruminal fluid samples using the TGuide S96 Magnetic Soil Stool DNA Kit (Tiangen Biotech) according to the manufacturer’s instructions. The hypervariable region V3–V4 of the bacterial 16S rRNA gene was amplified with primer pairs 338F: 5′-ACTCCTACGGGAGGCAGCA-3′and806R:5′-GGACT ACHVGGGTWTCTAAT-3′. PCR products were checked on agarose gel and purified through the Omega DNA purification kit (Omega). The purified PCR products were collected and the paired ends (2×250 bp) were performed on the Illumina Novaseq 6000 platform.

### Bioinformatic analysis

The qualified sequences with more than 97% similarity thresholds were allocated to one operational taxonomic unit (OTU) using USEARCH (version 10.0). Amplicon sequence variant (ASV) analysis using the DADA2 denoising algorithms. Taxonomy annotation of the OTUs/ASVs was performed based on the Naive Bayes classifier in QIIME2 [13] using the SILVA database [14] (release 138.1) with a confidence threshold of 70%. Alpha was performed to identify the complexity of species diversity of each sample utilizing QIIME2 software. Bacterial abundance and diversity were compared by one-way analysis of variance. The sequencing data were analyzed on the BMK Cloud online platform (https://www.biocloud.net).

### Metabolomics analysis and data processing

To comprehensively understand the metabolic changes in response to dietary energy levels, we employed LC-MS metabolomics. Analyses were performed using a Waters Acquity I-Class PLUS UPLC coupled with a Waters Xevo G2-XS QTof mass spectrometer. Chromatography used a Waters UPLC HSS T3 column with mobile phases of 0.1% formic acid in water and acetonitrile, under both positive and negative ion modes. This dual-mode approach allowed for comprehensive detection of a wide range of polar and non-polar metabolites, which is crucial for capturing the complex metabolic responses to dietary changes. Data preprocessing was done using Progenesis QI software, involving peak extraction, alignment, and identification with the METLIN database and an in-house library. The METLIN database was chosen for its extensive coverage of metabolites, which is essential for accurate compound identification. For data analysis, we normalized peak area data and used principal component analysis (PCA) and Spearman correlation to assess sample repeatability. Compounds were identified using the KEGG, HMDB, and lipid maps databases, which provide comprehensive pathway information. These databases were selected based on their relevance to metabolic pathway analysis and their reliability in providing accurate biological context.

Differential metabolites were determined using fold change, p-value, and VIP value from OPLS-DA modeling. This approach helped us identify key metabolites associated with different dietary energy levels, providing insights into the metabolic mechanisms underlying the observed physiological changes.

### Statistical analysis

Excel (Microsoft) was used for the preliminary processing of test data processing. SAS 9.2 was used for data variance analysis of data, and Student’s t-tests were used for multiple comparisons among different groups. The final test results were presented were expressed as the mean and standard error of the mean (SEM). p<0.05 indicated a significant difference and p<0.01 indicated a highly significant difference.

## RESULTS

### Growth performance

There was no significant difference in Initial weight, Final weight, ADG, ADFI, and F/G between the CON group and the HE group (p>0.05, Table 2).

### Reproductive performance

Table 3 shows the effects of different energy levels on the service of Holstein heifers at the initial service stage. While the HE group had higher estrus (95.83%) and conception rates (70%) than the CON group (91.43%, 55%), and a lower re-service rate (30% vs. 40% in the CON group), none of these differences reached statistical significance (p>0.05), suggesting that the trends may not be strong enough to support the hypothesis that energy levels significantly impact reproductive performance. However, the observed directional changes in these parameters could imply a potential influence of energy intake on reproductive outcomes that might become significant with a larger sample size or different experimental conditions.

### Reproductive hormones

Compared with the CON group, FSH, LH, and PRL in the HE group were significantly increased (p = 0.025, p = 0.041, p = 0.035, Table 4), while E2 and Prog levels showed no significant differences (p>0.05).

### Serum biochemical, immune, and antioxidant indexes

Compared with the CON group, the TG of the HE group was significantly increased (p = 0.024, Table 5), but other biochemical indexes were unaffected (p>0.05).

Compared with the CON group, IL-2 and IL-6 in the HE group were significantly increased (p = 0.001, p = 0.004, Table 5), and IL-4 was significantly decreased (p = 0.004), while the contents of TNF-α and IFN-γ were significantly decreased (p = 0.017, p = 0.032).

As shown in Table 5, MDA in the HE group was significantly lower than that in the CON group (p = 0.033), but other indexes showed no significant changes (p>0.05).

### Rumen fermentation parameters

The effects of different energy levels on the rumen fermentation parameters of Holstein heifers at the initial stage are shown in Table 6. Compared with the CON group, HE group cows had lower pH value (p = 0.010), and TVFA and acetic acid increased significantly (p = 0.002), the BCP, propionic acid, butyric acid, and valeric acid increased significantly (p = 0.027, p = 0.026, p = 0.040, p = 0.011). NH_3_-N, Isobutyric acid, Isovaleric acid, and the ratio of acetic acid to propionic acid showed no significant differences between the two groups (p>0.05).

### Rumen microflora

#### Rumen microbial diversity

**Figure 1:**
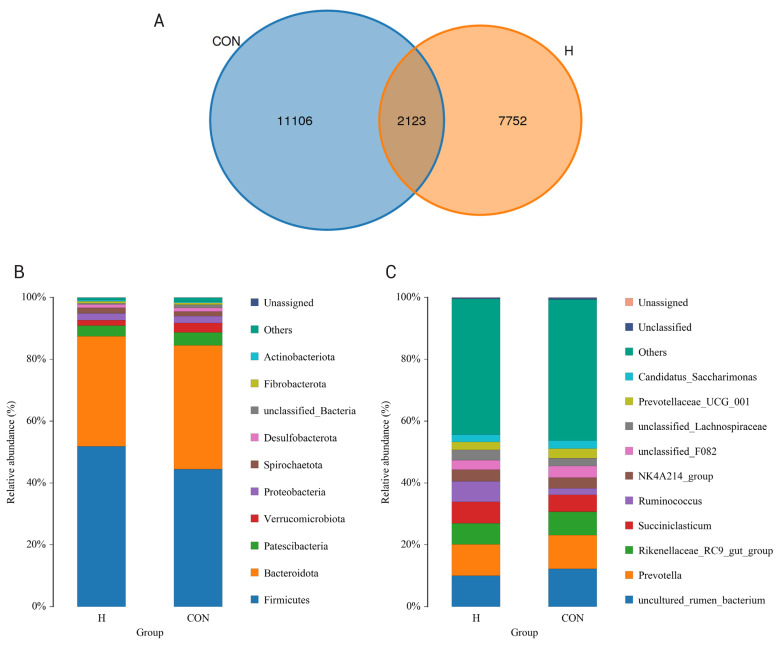
Effects of different energy levels on rumen microbial composition and communities. (A) Characteristic Venn diagram; (B) Distribution of main rumen bacteria at the phylum level; (C) Distribution of main rumen bacteria at the genus level.

As shown in Figure 1A, a total of 20,981 OTUs were produced in the rumen fluid of the two groups, of which 2,123 OTUs were shared. The HE group produced 9,875 OTUs, and the CON group produced 11,106 OTUs. The ACE, Chao1 and Simpson indexes were significantly lower than those in the CON group (p = 0.014, p = 0.014, p = 0.045, Table 7), while the Shannon index was also significantly lower (p = 0.001). The lower Shannon index may indicate reduced microbial diversity in the HE group, which could be a response to the altered dietary conditions and may have implications for rumen function and efficiency. However, the Simpson index’s approach to 1 suggests that the evenness of microbial communities was maintained, indicating that while diversity changed, the distribution of microbial taxa remained relatively stable.

#### Rumen microbial species composition

Rumen microbial composition is shown in Figures 1B, 1C. Compared with the CON group, the relative abundance of *Firmicutes* in the HE group was significantly higher than that in the control group (p = 0.038, Table 8), the relative abundance of *Verrucomicrobiota* was significantly lower than that in the CON group (p = 0.004). The increased *Firmicutes* abundance may be associated with the higher energy intake, as this phylum is often linked to more efficient energy extraction from the diet. The decrease in *Verrucomicrobiota*, although not significant in some contexts, could hint at shifts in specialized metabolic functions within the rumen microbial community that warrant further exploration in future studies with more focused hypotheses.

Compared with the CON group, the relative abundance of *Ruminococcus* in the HE group was significantly higher than that in the CON group (p = 0.018), *uncultured_rumen_bacterium* the relative abundance was significantly lower than that in the CON group (p = 0.006), *Prevotellaceae_UCG_001* was significantly lower than that in the CON group (p = 0.018). These taxonomic shifts, particularly the increase in *Ruminococcus*, which is known for its role in fiber degradation, may suggest that the HE diet is promoting the growth of microbes that can more effectively utilize the available energy substrates, potentially enhancing the overall efficiency of energy extraction in the rumen. However, the lack of significant differences in some cases may reflect the complexity of microbial interactions and the need for more nuanced approaches to understand these dynamics.

#### Rumen microbial metabolism

**Figure 2:**
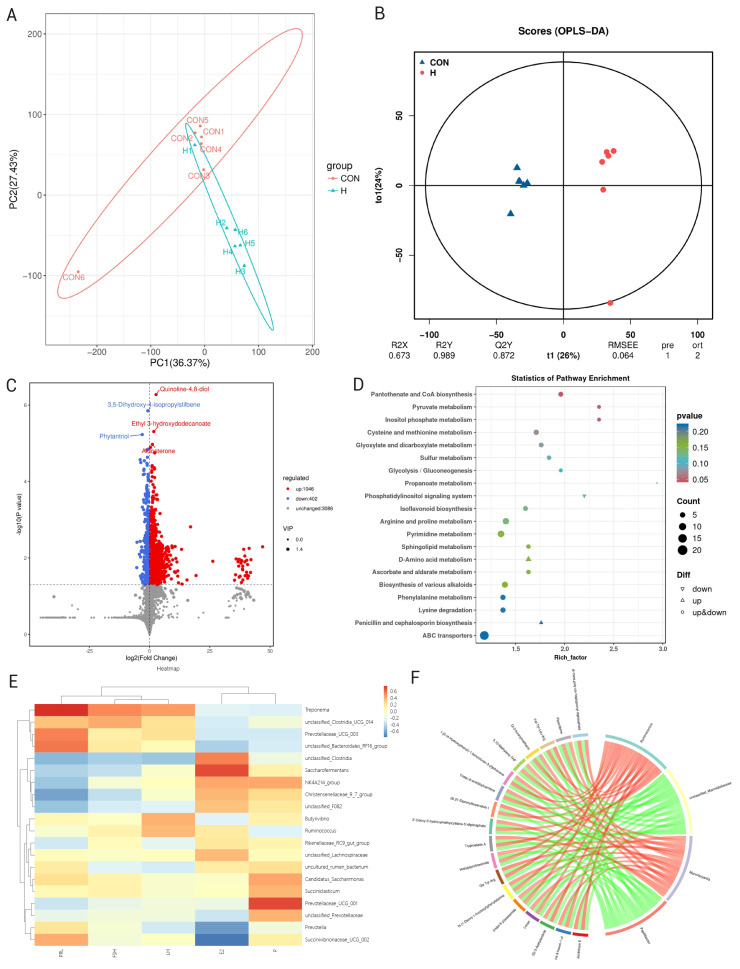
Effects of different energy levels on rumen metabolite profiles. (A) Principal component analysis diagram of differential grouping; (B) OPLS-DA score chart; (C) Differential metabolite volcanic diagram; (D) Differential metabolite KEGG enrichment map; (E) Microbial-reproductive hormones, correlation analysis heat map; (F) Differential metabolite-differential microbial correlation chord diagram. OPLS-DA, orthogonal partial least squares-discriminant analysis.

To compare compositional differences between the two groups, PCA was performed to reveal metabolic patterns. The PCA plot demonstrated a clear separation between the groups, indicating that different energy levels altered the rumen metabolomic profile (Figure 2A). Additionally, permutation tests were conducted to assess the validity and robustness of the OPLS-DA model, which showed R^2^Y = 0.989 and Q^2^ = 0.872. These metrics confirm the model’s effectiveness in explaining variability and predicting new data, ensuring it accurately represents the sample data (Figure 2B). As shown in Figure 2C, a comparison of CON and H identified 1448 differential metabolites (1,046 up-regulated and 402 down-regulated). Key metabolites, such as Quinoline-4,8-diol, Ethyl 3-hydroxydecanoate, Aldosterone, Phytantriol, and 3,5-Dihydroxy-4-isopropylstilbene, were significantly altered, indicating substantial changes in microbial metabolism and host physiological processes. KEGG pathway analysis revealed that the top 20 enriched pathways included Pantothenate and CoA biosynthesis, Pyruvate metabolism, and Inositol phosphate metabolism (The prominence of pyruvate metabolism in the enriched pathways is particularly noteworthy, as it aligns with the observed increase in TVFA and suggests that the HE diet is enhancing ruminal energy production through this key metabolic pathway, Figure 2D). This finding provides a potential mechanism linking dietary energy intake to microbial metabolic activity and ultimately to host energy status, although further research is needed to confirm the functional implications of these metabolic shifts.

### Correlation analysis

Cross-domain associations linking microbial community shifts to metabolite profiles (Figures 2E, 2F) demonstrated significant co-variation patterns across biological hierarchies. Correlation analysis of rumen microbial genera with reproductive hormones found that some rumen microbial groups were associated with hormones like PRL, FSH, LH, and P. The positive correlations observed between specific microbes and reproductive hormones suggest potential cross-talk between the rumen microbiota and host reproductive physiology. While these correlations do not prove causation, they highlight avenues for future research to explore how microbial modulation might influence reproductive outcomes, particularly in the context of different dietary energy regimes. For example, *Treponema* and *unclassified_Clostridia_UCG_014* were positively correlated with PRL, FSH, and LH, while *Saccarofermentans* were positively correlated with E2. *Prevotellaceae_UCG_001* was positively correlated with P. di-tians, poly-cis-heptaprenyl diphosphate, and Val-Tyr-Leu-Arg, which were in turn positively correlated with *Ruminococcus*. These correlations indicate that different energy levels can regulate the rumen microbial community and metabolism, which may have downstream effects on host physiological processes. However, the variability in these correlations and the lack of significant differences in some cases emphasize the need for caution in interpreting these results and suggest that additional factors or more precise experimental designs may be required to fully elucidate these relationships.

## DISCUSSION

Growth parameter evaluations revealed comparable outcomes for initial and final weights, ADG, ADFI, and F/G ratio between groups. This aligns with Williams et al [15]’s findings of similar results in growing heifers fed varying energy densities. It suggests Holstein heifers have adaptive mechanisms to maintain stable growth amid short-term dietary energy fluctuations. However, our study’s growth trend analysis was constrained by limited measurement time points (only days 1 and 21). This restricted our ability to capture detailed growth dynamics and potential anomalies. Future research should include more frequent measurements to better understand growth patterns.

In terms of reproductive performance, the estrus rate, conception rate, and pregnancy rate of the HE group were higher than those of the control group, but the difference was not significant. However, the FSH, LH, and prolactin in the HE group were significantly increased, while the E2 and Prog contents were not significantly different. FSH and LH are key regulators of the ovarian cycle, with FSH stimulating follicular development and LH triggering ovulation and luteinization. The significant increase in these hormones in the HE group suggests enhanced follicular maturation and ovulation efficiency, which may contribute to the observed trend of higher conception rates despite the lack of statistical significance. Additionally, PRL plays a crucial role in maintaining pregnancy by supporting corpus luteum function and Prog secretion. The elevation in PRL levels could indicate improved luteal phase maintenance, potentially supporting early pregnancy establishment. However, the lack of significant differences in estrus and conception rates may imply that these hormonal changes are not sufficient to drive significant improvements in reproductive outcomes within the study’s timeframe or that other factors are modulating these complex physiological processes. Further research is needed to explore the functional implications of these hormonal changes and their integration with other reproductive outcomes. This is consistent with the findings of Mellouk et al [16], who found that Holstein heifers fed a diet of different energy levels during the perinatal period had earlier onset of luteal activity after first calving in cows in the HE group, and the LH pulse frequency was higher than that in the low-energy group, suggesting that higher energy levels may potentially affect the reproductive performance of cows by modulating the secretion of reproductive-related hormones. While reproductive rate differences were not significant in this experiment, the hormonal changes imply that energy may regulate reproductive physiology, possibly under long-term or more extreme energy conditions. These results indicate a trend that could become significant with larger sample sizes or different experimental designs, and they provide a basis for future research to explore the underlying mechanisms of energy-reproductive hormone interactions.

In the serum biochemical indicators, TG were significantly increased in the HE group, which is consistent with the results of Yin [17], and Solomon et al [18]. Their study found that the serum TG content in the high and medium metabolic energy groups was significantly higher than that in the low-energy group. It may be that the appropriate increase in dietary energy levels increases the amount of energy and fat intake in the animal body, promoting the activation of the body’s fat transport mechanism, and resulting in serum TG content increases [19]. The balance of TG synthesis or metabolism is shifted towards synthesis, but there is no obvious damage or stress to the function of organs such as the liver. Other metabolic enzyme-related indicators of the liver are relatively stable, such as AST and ALT. The lack of significant differences in these liver enzymes, despite changes in TG, implies that the HE diet may be modulating lipid metabolism without causing detrimental effects on liver function.

Interleukin (IL-2, IL-6, IL-10) is a cytokine that interacts with leukocytes during the immune response process, and has the role of transmitting information, activating and regulating immune cells [20]. TNF-α and IFN-γ are innate immune-related cytokines with pro-inflammatory properties, which play a role in host defense, inducing inflammation and triggering apoptosis [21,22]. In terms of immune indexes, IL-2 and IL-6 in the HE group were significantly higher than those in the control group, IL-4 was significantly lower than that in the control group, and the content of TNF-α and IFN-γ was significantly lower than that in the control group. This shows that energy levels affect the immune regulation of cows. The results of Moyes et al [23] also show that the negative balance of energy and energy reduces the immunity of cows. This experiment found that HE diets may stimulate the immune response of the body increase the secretion of pro-inflammatory cytokines (IL-2, IL-6), and inhibit the secretion of some anti-inflammatory cytokines (IL-4) and immunomodulatory cytokines (TNF-α, IFN-γ). This change in immune status may be related to the regulation of energy in the body’s metabolism and stress response.

As a key marker of oxidative stress, MDA reflects the level and degree of lipid peroxidation in the body, and is one of the important indicators for evaluating the redox status of the body [24,25]. In the antioxidant index, the MDA in the HE group was significantly lower than that in the control group, this is consistent with the results of Chen et al [26], the MDA level gradually decreased with the increase of energy. There was no significant difference in GSH-Px, SOD, and T-AOC. MDA is a key indicator of oxidative stress, reflecting the level of lipid peroxidation in the body. The significant reduction in MDA suggests that HE diets may reduce oxidative stress, potentially benefiting the animals’ overall health. However, the lack of significant changes in antioxidant enzyme activities (GSH-Px, SOD) indicates that the body’s antioxidant defense system may not be significantly activated under the current energy levels. This could imply that the reduction in MDA is due to improved antioxidant efficiency or altered lipid metabolism rather than enhanced antioxidant enzyme activity. This indicates that HE diets lead to changes in lipid metabolism.

Studies have confirmed that the concentration of VFAs in the rumen and the ratio of acetic acid, propionic acid, and butyric acid are closely related to the type of feed and nutrient level. In a HE or concentrate-based diet, the concentration of VFA will increase significantly, with propionic acid increasing particularly significantly [27]. Rumen fermentation parameters showed that the rumen pH of the HE group was significantly lower than that of the control group, and the total VFAs and acetic acid were significantly higher than those of the control group. In Rabelo et al [28], it was found that the rumen pH of cows fed low-energy diets was higher than that of cows fed HE diets. Therefore, the higher the energy level in the diet, the lower the rumen pH [29]. The results of Xia et al [30] showed that the levels of ruminal bacterial protein, propionate, acetate, and total VFAs in cattle fed the HCP diet were significantly higher than those fed the LCP diet. Bacterial crude protein, propionic acid, butyric acid, and valeric acid were higher than those of the control group, the difference was significant. The lower pH value, higher TVFA, and acetic acid content showed that a HE diet promoted rumen fermentation, increased the accumulation of acidic substances in the rumen, and enhanced carbohydrate fermentation, which was related to the increase of substrate available to rumen microorganisms after the energy level was increased, and the changing of BCP and other indicators also reflected the changes of nutrient utilization and metabolism of rumen microorganisms and aligning with enhanced glucogenic potential in the HE group.

In terms of rumen microbial diversity, the ACE, Chao1, and Simpson indices of the HE group were significantly lower than those of the control group, and the Shannon index was significantly lower than those of the control group, indicating that the HE diet changed the diversity and richness of the rumen microbial community, which was consistent with Qiu et al [31]’s study on Holstein fattening cattle. The significant decrease in the Shannon index suggests a reduction in microbial diversity, which may indicate a more specialized microbial community rather than dysbiosis. HE diets may be select for specific taxa, such as *Ruminococcus*, that are better adapted to utilizing HE substrates, thereby enhancing the community’s specialization and efficiency in energy extraction. Therefore, appropriate dietary energy levels can ensure the normal growth and gastrointestinal health of ruminants [32].

At the phyla level, *Firmicutes* mainly produced acetate, propionate, and butyrate studies have shown that there is a relationship between *Firmicutes* and the body’s ability to obtain energy, and their ratio to *Bacteroidetes* is of great significance in maintaining homeostasis of the body and promoting the utilization of nutrients [33]. Specifically, the increase in the number of *Firmicute*s and the decrease in the number of *Bacteroidetes* contribute to the improvement of nutrient absorption efficiency, which has a positive impact on the body’s metabolic process, which has been fully demonstrated and reflected in previous relevant research results [34,35].

Nutritional interventions markedly altered microbial profiles, with HE-fed heifers exhibiting increased *Firmicutes* and decreased *Verrucomicrobiota* versus controls. Genus-level analysis revealed *Ruminococcus* enrichment, aligning with Park et al [36]’s findings of energy-driven taxonomic shifts across 23 genera. Functional predictions via PICRUSt2 confirmed metabolic pathway variations between dietary regimens.

*Ruminococcus* in the HE group was beneficial to the biosynthesis of amino acids in the rumen of dairy cows and the metabolism of pyruvate, glycerophospholipids, niacin, and niacinamide. These changes in microbial composition corresponded to changes in rumen fermentation parameters, such as *Firmicutes*, which is closely related to carbohydrate fermentation, and the 16.6% higher *Firmicutes* abundance in HE heifers (51.29% vs. 43.99%) aligns with enhanced fiber degradation capacity under HE diets, as evidenced by the 69.3% increase in total VFA production (60.47 vs. 35.73 mmol/L). This microbial shift likely contributes to the improved energy status driving gonadotropin secretion (LH +17.6%, FSH +21.2%), suggesting that farmers could prioritize energy-dense TMR formulations during the 12–14 month pre-breeding window to accelerate reproductive readiness. However, it is important to note that while these correlations provide valuable insights into potential host-microbe interactions, they do not establish causality. Future research could explore these relationships further using controlled experimental approaches such as functional assays or gnotobiotic models to validate the roles of specific microbial taxa in host physiological processes.

A large number of different metabolites were identified by LC-MS/MS metabolomics analysis, and the main enrichment pathways involved pantothenic acid and coenzyme A biosynthesis, pyruvate metabolism, and inositol phosphate metabolism. At the same time, correlation analysis showed that the rumen microbial community was closely related to metabolites, and some microorganisms were also related to reproductive hormones, such as *Treponema*, which was positively correlated with PRL, FSH, and LH. This further reveals the complex connection between rumen microbes, metabolism, and the overall physiological state of cows (including reproductive function). Energy levels affect the metabolic activities of microorganisms by changing the rumen microecological environment, which may indirectly regulate physiological processes such as reproduction in cows.

Based on current feed costs in Inner Mongolia, the 15% improvement in first-service conception rate (70%vs.55%) would reduce the average number of services per pregnancy from 1.82 to 1.43. For a 500-cow herd, this translates to 195 fewer inseminations annually, while increasing annual calf yield by 75 heads. While this study provides novel insights into energy-driven microbiota-reproduction interactions, the sample size (n = 22/group) and short trial duration (21 days) warrant caution in extrapolating long-term impacts. Future work should validate these findings across larger herds and diverse geographical settings, particularly in arid regions with limited feed resources.

## CONCLUSION

In conclusion, this study demonstrates that HE diets significantly altered reproductive hormone profiles (FSH, LH, PRL), rumen fermentation parameters (TVFA, pH, etc.), and microbial diversity (Shannon index, etc.) in primiparous Holstein heifers during the initial mating phase, while growth performance remained unaffected. These impacts are interconnected through an energy-modulated axis involving rumen microbiota shifts (e.g., *Firmicutes*), metabolic pathway activation (e.g., pyruvate metabolism), and endocrine regulation, collectively shaping physiological adaptation. Our findings provide empirical evidence for optimizing energy strategies in heifer rearing, though the period necessitates further validation of long-term reproductive outcomes. This work establishes a scientific framework for balancing productivity and metabolic health in dairy farming systems.

## Figures and Tables

**Table 1:** Ingredients and chemical composition of the basal diet

Item	CON	HE
Ingredient (% of DM)		
Straw kneading silk	33.45	32.75
Corn for silage	38.96	38.96
Corn	5.52	6.22
DDGS	4.14	4.14
Canola seed meal	7.86	7.86
Slowly-released urea	0.55	0.55
Premix^1)^	1.24	1.24
Corn spray protein	2.76	2.76
Mill offals	5.52	5.52
Total	100	100
Nutrient composition (%)		
DM	55.60	53.20
CP	12.90	13.80
EE	2.80	2.90
ASH	9.90	10.90
NDF	56.10	53.60
ADF	25.40	28.50
Ca	0.70	0.80
P	0.40	0.30
NE_L_ (MJ/kg)	8.64	9.50

1) The premix provided the following per kg of the diet: VA 180,000 IU, VD 65,000 IU, VE 1600 IU, Fe 1,850 mg, Cu 500 mg, Mn 2,260 mg, Zn 2,660 mg, Se 10 mg, I 21 mg, Co 18 mg.

DM, dry matter; HE, high-energy; DDGS, dried distillers grains with solubles; CP, crude protein; EE, ether extract; NDF, neutral detergent fiber; ADF, acid detergent fiber.

**Table 2:** Effects of different energy levels on the growth performance of Holstein heifers at the initial service stage

Items	CON group^1)^	HE group^2)^	SEM	p-value^3)^
Initial weight (kg)	404.45	392.47	5.31	0.109
Final weight (kg)	429.66	415.15	6.55	0.143
ADG (kg)	1.11	1.26	0.23	0.658
ADFI (kg)	15.13	15.44	0.25	0.380
F/G	6.31	7.14	1.28	0.654

1) Fed a basal diet.

2) Fed a high energy diet

3) Statistical significance was defined as p<0.05, and a highly significant difference was indicated by p<0.01.

SEM, standard error of the mean; ADG, average daily gain; ADFI, average daily feed intake; F/G, feed-to-gain ratio.

**Table 3:** Effects of different energy levels on the service of Holstein heifers at the initial service stage

Items	CON group^1)^	HE group^2)^	SEM	p-value^3)^
Number of estrous cows (head)	20	20	-	-
Estrus rate (%)	91.43	95.83	0.05	0.528
Conception rate (%)	55	70	0.10	0.320
Re-service rate (%)	40	30	0.07	0.356

1) Fed a basal diet.

2) Fed a high energy diet.

3) Statistical significance was defined as p<0.05, and a highly significant difference was indicated by p<0.01.

SEM, standard error of the mean.

**Table 4:** Effects of different energy levels on reproductive hormones of Holstein heifers at the initial service stage

Items	CON group^1)^	HE group^2)^	SEM	p-value^3)^
FSH (mIU/mL)	2.93	3.55	0.18	0.025
LH (mIU/mL)	7.68	9.03	0.41	0.041
PRL (μIU/mL)	161.24	187.13	7.84	0.035
E2 (pg/mL)	42.45	43.27	3.61	0.876
Prog (ng/mL)	46.66	42.63	4.66	0.577

1) Fed a basal diet.

2) Fed a high energy diet.

3) Statistical significance was defined as p<0.05, and a highly significant difference was indicated by p<0.01.

SEM, standard error of the mean; FSH, follicle-stimulating hormone; LH, luteinizing hormone; PRL, prolactin; E2, estradiol; Prog, progesterone.

**Table 5:** Effects of different energy levels on serum biochemical indexes of Holstein heifers at the initial service stage

Items	CON group^1)^	HE group^2)^	SEM	p-value^3)^
AST (U/L)	55.09	72.84	6.19	0.074
ALT (U/L)	24.62	29.17	2.47	0.236
ALP (U/L)	95.21	116.05	13.6	0.293
ALB (g/L)	28.38	33.24	2.63	0.210
TG (mmol/L)	0.23	0.30	0.02	0.024
TP (g/L)	57.45	70.85	5.06	0.077
LDH (U/L)	7.88	10.03	1.03	0.157
GLU (mmol/L)	4.18	4.74	0.28	0.178
Immune indexes	-	-	-	-
IL-2 (pg/mL)	242.87	312.97	13.19	0.001
IL-4 (pg/mL)	56.91	49.44	1.59	0.004
IL-6 (pg/mL)	156.16	184.33	5.97	0.004
TNF-α (pg/mL)	213.28	180.58	8.49	0.017
IFN-γ (pg/mL)	1,697.19	1,434.58	80.25	0.032
Antioxidant capacity	-	-	-	-
GSH-Px (U/mL)	384.63	424.74	21.66	0.235
SOD (U/mL)	82.74	84.71	1.99	0.506
T-AOC (mmol/mL)	0.32	0.35	0.01	0.062
MDA (nmol/mL)	2.49	2.03	0.14	0.033

1) Fed a basal diet.

2) Fed a high energy diet.

3) Statistical significance was defined as p<0.05, and a highly significant difference was indicated by p<0.01.

SEM, standard error of the mean; AST, aspartate aminotransferase; ALT, alanine aminotransferase; ALP, alkaline phosphatase; ALB, albumin; TG, triglycerides; TP, total protein; LDH, lactate dehydrogenase; GLU, glucose; IL, interleukin; TNF-α, tumor necrosis factor-α; IFN-γ, interferon-γ; GSH-Px, glutathione peroxidase; SOD, superoxide dismutase; T-AOC, total antioxidant capacity; MDA, malondialdehyde.

**Table 6:** Effects of different energy levels on rumen fermentation parameters of Holstein heifers at the initial service stage

Items	CON group^1)^	HE group^2)^	SEM	p-value^3)^
pH	6.88	6.38	0.11	0.010
NH_3_-N (mg/100 mL)	2.70	1.99	0.55	0.405
BCP (mg/100 mL)	9.19	11.73	0.65	0.027
TVFA (mmol/L)	35.73	60.47	4.35	0.002
Acetic acid (mmol/L)	24.76	41.72	2.85	0.002
Propionic acid (mmol/L)	6.36	12.46	1.66	0.026
Isobutyric acid (mmol/L)	0.34	0.37	0.04	0.635
Butyric acid (mmol/L)	3.41	4.81	0.42	0.040
Isovaleric acid (mmol/L)	0.48	0.60	0.08	0.302
Valeric acid (mmol/L)	0.38	0.51	0.03	0.011
The ratio of acetic acid to propionic acid	3.89	3.67	0.27	0.582

1) Fed a basal diet.

2) Fed a high energy diet.

3) Statistical significance was defined as p<0.05, and a highly significant difference was indicated by p<0.01.

SEM, standard error of the mean; NH_3_-N, ammonia nitrogen; BCP, bacterial crude protein; TVFA, total volatile fatty acid.

**Table 7:** Effects of different energy levels on the Alpha diversity index of rumen bacteria of Holstein heifers at the initial service stage

Items	CON group^1)^	HE group^2)^	SEM	p-value^3)^
ACE	2,752.52	2,185.75	133.92	0.014
Chao1	2,738.68	2,172.96	133.57	0.014
Simpson	0.997	0.996	0.000248	0.045
Shannon	9.91	9.52	0.06	0.001

1) Fed a basal diet.

2) Fed a high energy diet.

3) Statistical significance was defined as p<0.05, and a highly significant difference was indicated by p<0.01.

SEM, standard error of the mean.

**Table 8:** Effects of different energy levels on rumen bacteria at the phylum and genus levels of Holstein heifers at the initial service stage (%)

Items	CON group^1)^	HE group^2)^	SEM	p-value^3)^
*Firmicutes*	43.99	51.29	2.23	0.038
*Bacteroidota*	40.26	35.92	1.52	0.071
*Patescibacteria*	4.21	3.46	0.27	0.076
*Verrucomicrobiota*	3.07	1.76	0.25	0.004
*Proteobacteria*	2.34	2.24	0.47	0.879
*Spirochaetota*	1.53	1.77	0.20	0.397
*Desulfobacterota*	1.17	1.06	0.14	0.598
*unclassified_Bacteria*	1.04	0.40	0.30	0.164
*Fibrobacterota*	0.59	0.61	0.14	0.911
*Actinobacteriota*	0.39	0.45	0.15	0.789
Genus level				
*uncultured_rumen_bacterium*	12.25	10.02	0.46	0.006
*Prevotella*	10.89	10.10	0.85	0.523
*Rikenellaceae_RC9_gut_group*	7.61	6.89	0.60	0.413
*Succiniclasticum*	5.37	7.00	0.68	0.120
*Ruminococcus*	2.00	6.40	1.10	0.018
*NK4A214_group*	3.42	3.69	0.38	0.626
*unclassified_F082*	3.78	3.07	0.28	0.101
*unclassified_Lachnospiraceae*	2.49	3.29	0.45	0.238
*Prevotellaceae_UCG_001*	3.07	2.58	0.12	0.018
*Candidatus_Saccharimonas*	2.68	2.45	0.15	0.318

1) Fed a basal diet.

2) Fed a high energy diet.

3) Statistical significance was defined as p<0.05, and a highly significant difference was indicated by p<0.01.

SEM, standard error of the mean.
